# The School Doctor and the Mentally Defective Child
*The previous article appeared on May 27.


**Published:** 1916-06-10

**Authors:** 


### THE SCHOOL DOCTOR.

### The School Doctor and the Mentally Defective Child (*continued*).*

The history of day special schools in this country since their institution in Leicester and London in 1892 has been a record of increasing rise in the public confidence. Certain extravagant hopes of their possibilities in curing subnormal mentality have been abandoned, but only to be replaced by a wise appreciation of their true function of making the best of Nature's bad jobs. Most of all, perhaps, does the school doctor value a very clearly discerned change of attitude among the parents themselves, who are increasingly inclined to regard special education for their children as a privilege rather than a penalty.

Such popular prejudice as remains is due not so much to resentment against the " stigma " as to that section of the Defective and Epileptic Children Act which empowers local authorities to retain children in special schools until the age of sixteen. The advantages of this provision are mainly theoretical, the hardships inflicted on the parents and children themselves are very real, and the working of the Act runs most smoothly where children are wisely allowed to leave at any time after fourteen, provided suitable work has been found for them.

Where " general intelligence " and scholastic attainments show an approximately equal amount of retardation the doctor's decision is usually accepted; difficulties begin where the intelligence is very little below normal, and yet in spite of good teaching and regular attendance the child proves incapable of progress in school subjects.

Formerly these disputed cases had to be fought out before a magistrate, but now, fortunately, they have to be referred to the Board of Education, whose decision is binding. Two types predominate among these borderline cases. Firstly, the " superficially bright " child, alert, friendly, self-possessed, a sharp observer, a good talker, often an amusing mimic, and yet utterly incapable of progressing in any school subject. These little ones are usually as bad at handwork as at the " three R's," and the prognosis in their case is unsatisfactory. Towards adolescence the veneer of brightness rubs off and reveals the essentially poor quality of the mind, but during childhood the only psychological weakness visible is a lack of concentration. They are none the less " defective " within the meaning of the Act, and if allowed to remain in the ordinary school they may attain the age of ten or twelve without being able to spell " cat " or to give change out of sixpence.

The second type is the dull, slow, bucolic child, possessed of few interests and a very limited range of ideas, but capable of using those ideas to considerable advantage. They are often good (though not so good as is commonly supposed) at manual work, and they do well in after-life provided that fate puts no heavy strain on their judgment or resource. The provision of " intermediate " schools or classes is desirable for many of these boys and girls, but, failing these, the children's own interests demand their transfer from the ordinary school, where sheer boredom over unsuitable book-work drives them into vicious ways, to the special school, where the training is suited to their capacities.

The nervous type of defective child gives rise to another series of problems and misconceptions on the part of the public. The presence of any one of those multifarious symptoms of motor excitability or emotional disturbance which we loosely class as " nervousness " is invariably seized on by the parents as an excuse for the child's backwardness, however low its intelligence may be. Every school doctor has seen cases where defective children have been kept out of school for years (and have incidentally sustained irreparable mental damage) on medical certificates of " neurasthenia " or " nervous overstrain," and it is extraordinarily difficult to persuade parents, or even teachers, that " nervousness " may be the effect, or at least the accompaniment, rather than the cause, of feeble-mindedness. Experience confirms the view that symptoms such as tics, nerve storms, fits of sobbing, night terrors, are greatly augmented by the noisy bustle of a large school and the strain of hopeless competition with mental superiors. The appropriate treatment is not exclusion but the quiet atmosphere of a special school where the over-wrought child can compete with its fellows on equal terms and recover its self-respect. It is hardly too strong to say that defectives of nervous tendencies never suffer, but rather benefit, from the presence of children " worse than themselves."

A group of children who bring the doctor into occasional conflict with the teachers are those in whom the " mental defect " is shown by abnormalities of conduct and morals rather than by inability to learn. They may be perhaps a standard or two behind their fellows; their general intelligence is fairly good, though rarely up to the average. It is impossible to call these children mentally normal; they are irrational and senseless in their misdeeds, they show little response to reward or punishment, they are often very deficient in inhibition and liable to maniacal outbursts of temper; yet they are clearly unsuited to a day special school. The legal definition which makes inability to progress in the elementary school the qualification for admission puts that out of the question. Furthermore, the slow rate of work set by genuine defectives would leave them with abundant time for mischief, the weak, suggestible character of defectives providing ample scope for contamination. Certification as " moral imbeciles " for the worst delinquents, industrial schools for the law-breakers, and firm but sympathetic discipline in the ordinary school for the remainder, are the only solutions at present available.

* The previous article appeared on May 27.

